# Dietary Flexibility of Calanoid Copepods in the Sub‐Arctic Atlantic: The Role of Protistan Microzooplankton

**DOI:** 10.1002/ece3.71080

**Published:** 2025-03-16

**Authors:** Elliott Price, Claire Mahaffey, Rowena Stern, Claudia Castellani, Rachel M. Jeffreys

**Affiliations:** ^1^ School of Environmental Sciences University of Liverpool Liverpool UK; ^2^ Tiny Ocean Health Insights Ltd Plymouth UK; ^3^ Stazione Zoologica Anton Dohrn Napoli Italy

**Keywords:** amino acids, Arctic, Barents Sea, *Calanus*, compound‐specific stable isotopes, trophic position

## Abstract

Zooplankton play a key role in marine food webs, transferring energy from the base of the food web to higher trophic levels. In the Arctic, warming is altering nutrient availability and primary productivity, which could alter zooplankton‐mediated transfer of energy through food webs. The Barents Sea Opening is warming rapidly, and has a strong influence on the Arctic as it is a prominent gateway for North Atlantic water advected into the polar region. Trophic position (TP) is an important metric because it identifies the location of an organism within a food web and therefore provides insight on food web functioning. Using nitrogen isotopes of amino acids in copepods, we investigated how the food web baseline and TP of the keystone *Calanus* species change in response to environmental gradients along the Barents Sea Opening in summer between 2010 and 2016. Spatial and interannual variation in net primary production and the North Atlantic Oscillation index both strongly influenced the nitrogen isotope baseline. We demonstrate that protistan microzooplankton play a key role in the diets of *Calanus* spp., accounting for 1–2 TP steps determined using alanine (TP_Ala_) and that this varied spatially and interannually; however, the TP of *Calanus* spp. determined using glutamic acid (TP_Glu_ = 2.2 ± 0.2) indicated consistent herbivorous feeding. Flexibility in the diet of *Calanus* spp. under differing environmental conditions suggests that *Calanus* spp. may be able to adapt to changing food availability created by environmental instability driven by climate change.

## Graphical abstract

Zooplankton play a key role in marine Arctic food webs, transferring energy from the base of the food web to higher trophic levels. Here we show spatial and interannual variation in net primary production and the North Atlantic Oscillation index in the Barents Sea Opening, which strongly influenced the nitrogen isotope baseline. We also show that *Calanus* copepods rely on both protistan microzooplankton and phytoplankton. *Calanus* spp. reliance on the protistan food web varied spatially and temporally whilst maintaining a high degree of herbivory. Thus, protistan microzooplankton may offer an alternative food source for *Calanus* spp. when the availability of phytoplankton becomes limited.

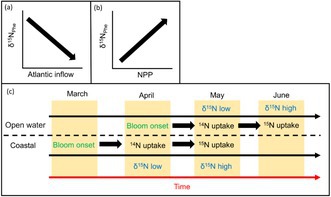

## 1 Introduction

Zooplankton occupy intermediate trophic levels in marine food webs. They bridge the transfer of energy produced at the base of the food web to larger organisms at higher trophic levels (Falk‐Petersen et al. 2009). Fundamental characteristics of zooplankton biogeography, behaviour, and life history and phenology are being altered across the globe due to climate change (Bollens et al. 2002; Mackas et al. 2012; McGinty et al. 2021; Richardson 2008). The intrusion of zooplankton into the habitat of other taxa resulting from shifting distributions (Beaugrand et al. 2009) increases local competition for food resources (Alley 1982). In addition, regional changes in net primary production (NPP) (Gregg and Rousseaux 2019) and phytoplankton community structure (Finkel et al. 2010; Huertas et al. 2011) across the global ocean could lead to reductions in zooplankton fitness if the new food environment is less nutritious (Bi and Sommer 2020; Prince et al. 2006). Widespread changes to zooplankton communities across the global ocean will propagate into the wider food web. These impacts have implications for the survival of endangered, vulnerable, and threatened species and the sustainability of fisheries, both of which depend on stable and healthy populations of zooplankton (Beaugrand and Kirby 2010; Laidre et al. 2008).

Zooplankton species associated with sub‐Arctic and temperate regions, such as the copepod *Calanus finmarchicus* , are expanding their distributions further north into the Arctic (Freer et al. 2022; Møller and Nielsen 2019; Reygondeau and Beaugrand 2011). This species is advected into the Arctic through the Barents Sea Opening and the eastern Fram Strait (Daase et al. 2007) and is the dominant zooplankton taxon in these two regions (Aarflot et al. 2018; Carstensen et al. 2019). However, the extent of its advection into the Arctic is increasing since the Barents Sea is currently undergoing long‐term change in a process known as ‘Atlantification’ (Årthun et al. 2012; de la Vega et al. 2022). In the southern and western Barents Sea, the volume of Atlantic water has more than doubled in the last 30 years (Oziel et al. 2016), and *C. finmarchicus* is replacing the endemic Arctic species *C. glacialis* and *C. hyperboreus* (Aarflot et al. 2018). A positive phase of the North Atlantic Oscillation (NAO) exacerbates ongoing Atlantification (Koenigk and Brodeau 2014), leading to warmer temperatures that modify conditions in the region, including changing mixed layer depths, which ultimately impact nutrient delivery to the surface waters and alter NPP (Buchanan et al. 2022; Tuerena et al. 2021a).

Atlantification of the Arctic has altered the timing and intensity of primary and secondary production (Leu et al. 2011; Lewis et al. 2020), with implications for zooplankton dynamics. As a result, there is a mismatch between organisms and their food supply, with implications for the reproduction, development, and survival of marine organisms at all levels of the food web (Kaartvedt and Titelman 2018; Laidre et al. 2008; Régnier et al. 2019; Stige et al. 2019). *Calanus* spp. convert carbon from primary producers into their lipid sacs (Lee 1975; Falk‐Petersen et al. 1987), forming a dense energy source. Calanoid lipids are in part transferred up the food chain and are a major source of energy for the many marine predators that feed extensively on *Calanus* spp., including fish (Olsen et al. 2010), seals (Falk‐Petersen et al. 2009), whales (Blanchet et al. 2019) and seabirds (Jakubas et al. 2017). Therefore, any reduction in *Calanus* spp. will have detrimental consequences for higher trophic levels and the marine pelagic ecosystem (Varpe et al. 2005). Recent work has shown that Arctic zooplankton communities are changing, as the boreal *Calanus finmarchicus* can now complete its lifecycle in the Arctic (Tarling et al. 2022). Boreal *C. finmarchicus* has benefitted from the long‐term retreat of the ice edge and subsequent changes in phytoplankton phenology, taking advantage of a longer feeding period (Freer et al. 2022; Tarling et al. 2022). Changes to zooplankton communities will likely have consequences for food web function in the Arctic (Tarling et al. 2022).

*Calanus* spp. are opportunistic omnivores that mitigate shortages in the food environment by compensatory feeding on secondary producers such as ciliates and heterotrophic flagellates, microzooplankton, eggs, and developmental stages of calanoid copepods (Frank‐Gopolos et al. 2017; Levinsen et al. 2000; Ohman and Runge 1994), which could lead to a measurable increase in their trophic position (TP). Whilst compensatory feeding through switching to feed on a different source of phytoplankton (Søreide et al. 2008) would lead to no change in TP. The nutritional value of phytoplankton varies between species (Irigoien et al. 2000; Leiknes et al. 2014), so if *Calanus* spp. switched to feed on less nutritious phytoplankton, they must spend more time feeding to support their physiological demands, leaving them vulnerable to predation for extended periods (Hobbs et al. 2018). Additionally, a switch to a more omnivorous diet would increase the length of the food chain, which would reduce the energy efficiency of the food chain, as *~*85% of energy is lost during transfer at each trophic level (Baumann 1995). This loss of energy could lead to large differences in biomass production for the entire system (Eddy et al. 2021).

To investigate the TP of *Calanus* spp. in relation to changing environmental conditions in the Barents Sea, we analysed compound‐specific nitrogen isotope values of individual amino acids (CSI‐AA) from specimens collected by the Continuous Plankton Recorder (CPR) Survey in the Barents Sea Opening from June over a period of 6 years, between 2010 and 2016. CSI‐AA of nitrogen isotopes allows for the identification of TP due to the fractionation/conservation of nitrogen isotopes in certain amino acids between trophic levels (McClelland and Montoya 2002). ‘Trophic’ amino acids, for example, glutamic acid and alanine, are heavily fractionated between trophic levels, whilst ‘source’ amino acids, for example, phenylalanine, represent the base of the food web, as minimal isotopic fractionation during assimilation means the δ^15^N of phenylalanine (δ^15^N_Phe_) of the resource is conserved in the tissues of the consumer (McClelland and Montoya 2002). Source AAs can be used to trace environmental changes at the base of the food web. For example, the δ^15^N of nitrate (δ^15^NO_3_) is integrated into source AA isotope ratios via phytoplankton (Espinasse et al. 2019; Tuerena et al. 2021b), as are the δ^15^NO_3_ signals of different water masses (McClelland et al. 2003; Mompeán et al. 2016), such as those of Pacific and Atlantic waters (Buchanan et al. 2022; de la Vega et al. 2021a; Tuerena et al. 2021b), or coastal versus open water (Thibodeau et al. 2017). TP estimates can be determined using the difference between the δ^15^N in the trophic and source amino acids, divided by the δ^15^N enrichment at each trophic step (Chikaraishi et al. 2009; Nielsen et al. 2015). Glutamic acid is known to lead to TP estimates representing only the metazoan steps in food webs and may underestimate a consumer's TP, whereas using alanine to estimate TP accounts for both metazoan and protistan steps in food webs (Gutiérrez‐Rodríguez et al. 2014; Décima et al. 2017; Décima et al. 2020).

**FIGURE 1:**
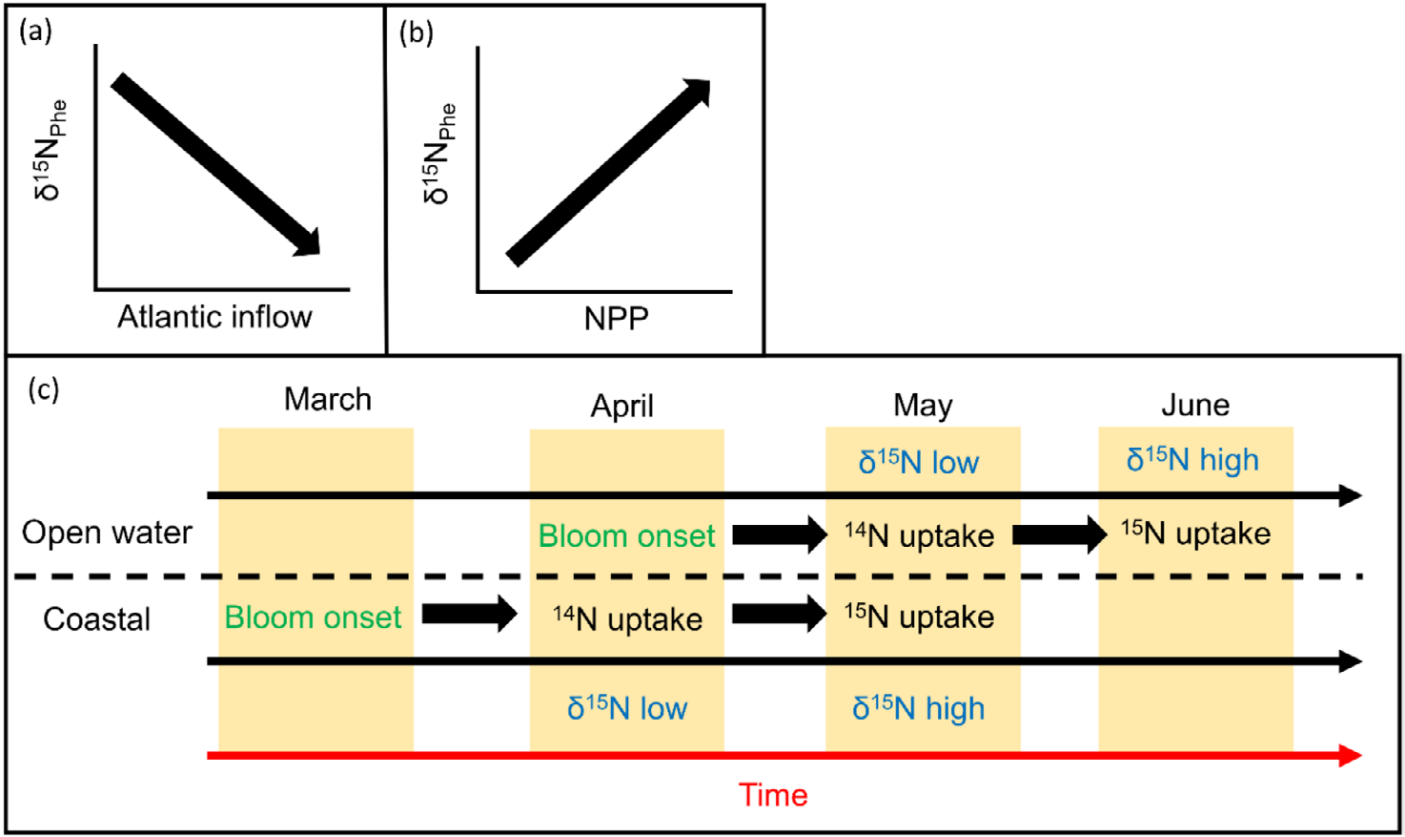
Schematic depicting predictions for the evolution of the nitrogen isotopic baseline in the Barents Sea Opening (δ^15^N_Phe_) with (a) the amount of Atlantic water in flowing into the region, and (b) net primary production. The potential mechanism for differences in δ^15^N values between coastal and open water regions are illustrated in (c). ‘Uptake’ refers to the assimilation of the N isotope by phytoplankton.

The aim of this study was to determine: (1) how the TP of *Calanus* spp. varies on annual scales and along environmental gradients across the Barents Sea Opening and (2) if variation in the TP of *Calanus* spp. is driven by variation in the δ^15^N baseline (δ^15^N_Phe_). We hypothesise that δ^15^N_Phe_ will vary spatially between coastal and open water regions due to differences in bloom phenology (Figure 1), whilst temporal variability in δ^15^N_Phe_ will be associated with changes to NPP (Figure 1). We also expect that variability in the TP of *Calanus* spp. will be linked to spatial and interannual differences in NPP, and that lower levels of NPP will lead to increased TP as they utilise a more omnivorous feeding strategy to compensate for a reduction in phytoplankton food availability.

## 2 Materials and Methods

### 2.1 Study Area

**FIGURE 2:**
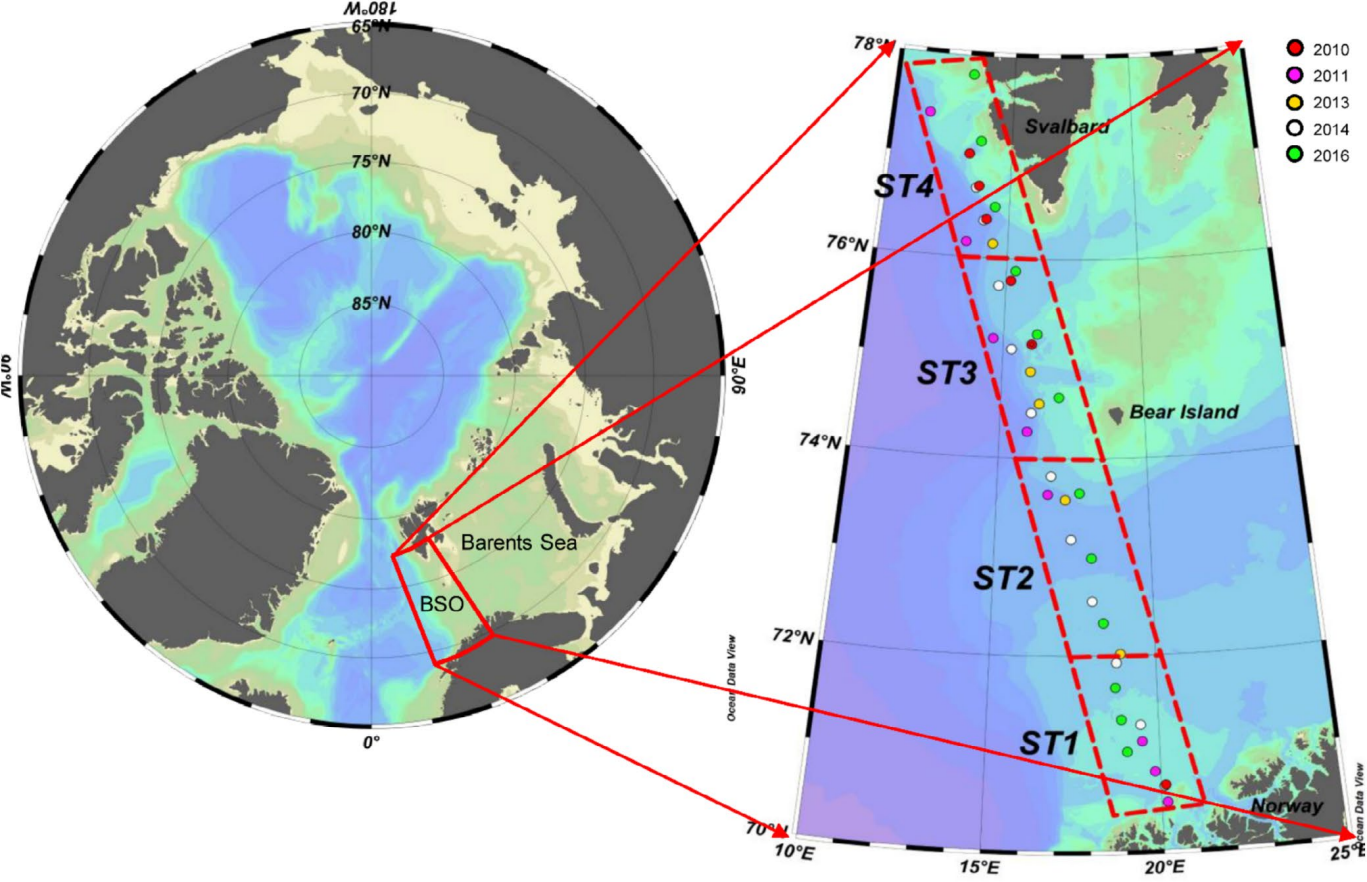
Sample locations of the Continuous Plankton Recorder Survey time series across the Barents Sea Opening used for compound‐specific stable isotope analysis of amino acids in *Calanus* spp. copepods. Coloured dots represent the different years of sampling. Red boxes denote the regions of the transect (ST1—ST4).

We focused on the northernmost route of the Continuous Plankton Recorder time series, which traverses the Barents Sea Opening (BSO) between Tromsø (70°N, 20°E) and Svalbard (78°N, 12°E) (Figure 2). The Svalbard–Tromsø (ST) route covers a sub‐Arctic Atlantic gateway in the southwestern Barents Sea where warm (T > 3°C) and saline (S > 35 PSU) North Atlantic water masses are advected north of and into the central Barents Sea shelf, and cold/fresh waters (T < 0°C/S < 34.7 PSU) are advected south around the northern edge of Bear Island (Barton et al. 2018). The two water masses meet, creating a frontal zone at 74°N along the transect (Barton et al. 2018). The water column depth of the main ST route is between 100 and 400 m, with some samples from 2011 being collected on the western continental slope at a maximum depth of 1150 m.

The ST transect covers a hydrologically diverse region in terms of bathymetry and water masses (Figures S1 and S2). We separated the transect into four regions representing different water masses based on average SST and salinity values across the time series. These regions shown in Figure 2, comprised of: (1) ST1: the southern coastal region between 70°–72°N, which is influenced by the fresher Norwegian Coastal Current (NCC; Figure S2); (2) ST2: 72°–74°N region, which is away from the coastal influence, and where warm saline waters enter from the north Atlantic (Figure S2); (3) ST3: The 74°–76°N region that is situated parallel to the polar front that is shaped by the bathymetry and hydrology surrounding Bear Island (Figures S1 and S2); (4) ST4: 76°–78°N which covers areas immediately to the southeast of Svalbard (Figures S1 and S2). We grouped ST2, ST3 and ST4 together as the ‘open water region’ and we refer to ST1 as the ‘coastal region’ based on the influence of the fresh and warm Norwegian Coastal Current that influences plankton food web phenology (Skjoldal et al. 2021) and due to the similarity in their hydrographic and water mass properties (Tuerena et al. 2021b; Figure S1).

### 2.2 Plankton Sampling and Selection

Plankton samples were collected along the ST route using the CPR, towed behind ships of opportunity at a depth of *~*10 m (Richardson et al. 2006). Plankton are collected on moveable bands of silks and preserved in a 4% formalin solution. The silks are then cut into segments that each represent 10 nautical miles of the ocean surface along the transect; each segment was treated as a single CPR sample where phytoplankton and zooplankton individuals are preserved, and taxa and life cycle stages are recorded (Richardson et al. 2006). We selected individual adult female and CV stages of *Calanus* spp. from samples collected in June between the years 2010 and 2016, excluding 2012 as no sampling was conducted and excluding 2015 as sampling was only conducted in ST1 (Table 1; Figure 2). Sampling effort in June was the most consistent on a spatial and annual scale across the CPR time series for the ST route. Additionally, June coincides with high zooplankton abundance post‐spring bloom (Dalpadado et al. 2020; Hassel 1986; Verity et al. 2002), offering a good representation of *Calanus* spp. populations in the region across the time series. For most CPR samples, one pool of between 20 and 50 individual copepods that morphologically resembled *Calanus finmarchicus* was picked (number of samples with one pool = 48), and two pools of 50 individuals were picked where copepod abundance was ≥ 100 individuals. Where two pools of samples were available, these were treated as duplicates of the same sample and not treated as statistically independent. Distinction was made between *Calanus hyperboreus* and *C. finmarchicus* /*glacialis* based on prosome length: > 5 mm ( *C. hyperboreus* ), < 4 mm *C. glacialis* /*finmarchicus*. Community composition data from the CPR survey for this region suggest that a majority of the *Calanus* copepods are *C. finmarchicus* rather than *C. glacialis* (Strand et al. 2020); therefore, we are confident that most of the individuals used in the present study are *C. finmarchicus* . However, molecular analysis was not used to confirm identification, so there may be inclusion of *Calanus glacialis* and *Calanus helgolandicus* , and so will be referred to as *Calanus* spp. from here on. Copepods were stored in 0.5 mL of a 4% formalin solution at room temperature until analysis.

**TABLE 1:** Number of separate pools of *Calanus* spp. for each year and each region of the Barents Sea Opening used for stable isotope analysis of phenylalanine, a source amino acid and alanine and glutamic acid, trophic amino acids. Each pool consists of between 20 and 50 *Calanus* individuals picked from each CPR sample. Each CPR sample is a segment of silk that represents 10 nautical miles of the ocean surface along the ST transect.

		70°–72°N	72°–74°N	74°–76°N	76°–78°N
Trophic amino acids: Alanine and Glutamic acid	2010	1	0	2	3
	2011	8	3	3	3
	2013	0	4	4	1
	2014	8	4	5	4
	2016	4	5	4	3
Source amino acid: Phenylalanine	2010	1	0	2	3
	2011	7	3	3	2
	2013	0	4	4	1
	2014	7	4	5	4
	2016	3	5	3	3

### 2.3 δ^15^N Isotope Analysis of Amino Acids

Previous work on similar time‐series samples for zooplankton has indicated that the effect of formalin on the δ^15^N values of AAs is minimal (Hannides et al. 2009; Hetherington et al. 2019). However, Hetherington et al. (2019) observed a 3‰ enrichment in the δ^15^N value of phenylalanine over a 25‐year period in two copepod species; this was due to coelution of phenylalanine with other N‐containing compounds, which we did not observe in our samples. Furthermore, we rinsed our samples thoroughly in Milli‐Q water prior to analysis to remove any phytoplankton or debris attached to the copepods and to rinse off residual formalin.

Therefore, to our knowledge, the δ^15^N of amino acids used to calculate TP would be equally modified by formalin preservation and thus have a limited impact on our TP estimates.

CSIA‐AA of *Calanus* spp. samples was carried out at LIFER (Liverpool Isotopes for Environmental Research Laboratory) at the University of Liverpool following the methods of Corr et al. (2007). Copepod samples were lyophilised and homogenised using a pestle and mortar. Sample biomass availability was variable, and a minimum of 1 mg of tissue is required for analysis, but generally *~*15 mg of sample was utilised. Samples were hydrolysed in reaction vessels (6 M HCl, 300 μL, 110°C, 22 h). An internal standard, L‐Norluecine (Sigma‐Aldrich) was added to each sample (30–75 μL at 5000 ng mL^−1^ depending on sample mass) prior to hydrolysis. Once cool, the hydrolysate was filtered using a nanosep centrifugal device (0.2 μm PTFE filter) to remove salts and impurities. Lipids were extracted via vortex (n‐hexane:DCM, 3:2 v/v, 2 mL) and removed from the hydrolysate supernatant. The lipid‐extracted hydrolysate was frozen at −80°C and then lyophilised overnight.

Amino acids were propylated with 0.25 mL of isopropanol: acetyl chloride (4:1, v/v) over an ice bath and heated (100°C, 1 h). Propylates were placed in a freezer (−20°C) for 5 min to quench the reaction, and reagents were removed under a gentle stream of N_2_ gas. DCM (1 mL) was added (×3) and evaporated to ensure the removal of excess reagents. Acetylation was carried out through the addition of 1 mL of an acetone:triethylamine:acetic anhydride (5:2:1, v/v), heated (60°C, 10 min). Following this, reagents were removed under a gentle stream of N_2(g)_, and the samples were re‐dissolved in 2 mL of ethyl acetate; 1 mL of saturated NaCl solution was added. Phase separation was enabled by mixing, and the organic phase was collected and passed through a column containing MgSO_4_ (removal of water); this was repeated 3 times. The filtered derivatised amino acid fraction was dried under a gentle stream of N_2_. The derivatised amino acids were redissolved in DCM and stored at −20°C until analysis via GC‐IRMS.

δ^15^N_AA_ values were measured using a Trace Ultra gas chromatograph (GC) coupled to a Delta V Advantage IRMS with a ConFlo IV interface (Cu/Ni combustion reactor held at 1000°C, Thermo Fisher). A liquid nitrogen trap removes CO_2_ from the sample stream, situated after the reduction oven. Amino acids were separated using an HP Innowax capillary column (30 m × 0.25 mm i.d. × 0.5 μm film thickness, Agilent). Samples were introduced to the column using a split/splitless injector set at 260°C. The GC oven program was: 50°C, 2 min, 10°C min^−1^ to 180°C and 6°C min^−1^ to 260°C, hold 16.7 min. The carrier gas was ultra‐high purity helium (flow 1.1 mL min^−1^). The ion intensities of m/z 28, 29, and 30 were monitored, and the δ^15^N of each amino acid peak was automatically computed (Isodat version 3.0; Thermo fisher) by comparison with a standard reference N_2_ gas, which was measured at the beginning (×4) and end (×4) of each sample analysis.

All results are reported in per mil (‰) relative to atmospheric N_2_ (=0‰). Each sample was analysed in duplicate, and where the standard deviation was > 1 between the two duplicate δ^15^NAA measurements, the sample was analysed again in duplicate. Precision and accuracy of measurements were determined using a mixed amino acid standard prepared from 7 amino acids with known δ^15^N values, including: alanine, valine, leucine, aspartic acid, glutamic acid, glycine, and phenylalanine (University of Indiana, USA and SI Science Japan). The mixed standard was analysed every four injections, and the accuracy and the precision of all mixed standard injections are summarised in Table S1.

Measured δ^15^NAA sample values were corrected following McCarthy et al. (2013), Equation 1. This method takes into consideration the response of individual amino acids to the stationary phase of the column and is based on the offset between the measured δ^15^NAA values in the nearest mixed standard and their known δ^15^NAA values Equation 1.

(1) $$\begin{array}{cc} \delta^{15}N-{\text{Sample}}_{\text{reported}}= & \;\mathrm{Avg}\;\delta^{15}N-{\text{Sample}}_{\text{measured}}\\ & \;-\left(\delta^{15}N-{\text{Standard}}_{\text{measured}}-\delta^{15}N_{\text{known}}\right) \end{array}$$

where Avg δ^15^N‐Sample_measured_ is the average δ^15^N for an amino acid in a sample (*n* = 2), δ^15^N‐Standard_measured_ is the δ^15^N for the AA in the nearest mixed standard, and δ^15^N_known_ the known elemental analysed offline value for the same standard. Results are reported in δ‐notation (‰), relative to atmospheric N_2_.

### 2.4 Environmental Variables

A suite of environmental variables was selected to identify potential drivers in the δ^15^N_Phe_ and in the relative TP of *Calanus* spp. Detailed descriptions of the resources and calculations used to obtain the following variables and the limitations of this approach can be found in Data S2. Sea surface temperature (SST) and surface salinity were selected as they reflect different water masses (Tuerena et al. 2021b), and the North Atlantic Oscillation index was used as a proxy for short‐term increases in Atlantic inflow into the Barents Sea (Koenigk and Brodeau 2014). NPP and total phytoplankton carbon were used to represent the food environment for *Calanus* spp. Environmental variables related to known mechanisms that influence the nitrogen isotope baseline were selected to identify potential drivers; these included NPP, total phytoplankton carbon, nitrate and phosphate concentration, and N* (Gruber and Sarmiento 1997), used as a water mass tracer for this region (Tuerena et al. 2021b).

### 2.5 Data Processing and Analysis

All statistics and data processing was carried out in R Studio (R Development Core Team 2024). NetCDF files of the various environmental variables were downloaded (Data S2) and unpacked using the ‘ncdf4’ package in R. The gridded environmental datasets produced were then matched to each CPR sample by latitude and longitude, and average monthly values at each CPR sampling location were calculated for April, May, and June to capture present and lagged effects of the environment on the isotope measurements. Further evaluation of temporal trends in SST, NO_3_ concentrations, NPP, phytoplankton carbon, N*, and the North Atlantic Oscillation index was performed using time‐series decomposition to effectively eliminate seasonality from the dataset, revealing underlying temporal patterns. Subsequently, various models were fit to statistically evaluate the underlying patterns, including linear models and polynomial models (quadratic and cubic), and evaluated the outputs for best model fit.

To identify spatial and interannual variability in the δ^15^N_Phe_ baseline, we used a one‐way Analysis of Variance (ANOVA) and Tukey's HSD post hoc tests between the three regions within the open water grouping and between each year (year was treated as a factor). The residuals of each ANOVA model were checked for normality to assess the model's fit. An unpaired *t*‐test identified differences in the δ^15^N_Phe_ between the open water and coastal groups.

We used linear regressions (LR) to test the hypothesis that the δ^15^N_Phe_ was driven by NPP, N*, phytoplankton carbon, and nutrient concentrations. The relationship between δ^15^N_Phe_ and salinity and SST was used to identify nitrogen isotope baseline patterns in different water masses. We assessed the correlation between δ^15^N_Phe_ and NPP, total phytoplankton carbon, concentrations of nitrate, phosphate, and N* for April, May, and June to identify drivers of δ^15^N_Phe_ interannual variability in the open water region. In addition, we used a positive North Atlantic Oscillation index as a proxy for increased Atlantic water intrusion in the Barents Sea (Koenigk and Brodeau 2014) and analyzed its correlation with δ^15^N_Phe_ a linear regression model.

The TP of *Calanus* spp. reflects their functional feeding habit within a food web, where for *Calanus* feeding exclusively on phytoplankton, we would expect a TP of 2 reflecting true herbivory, a TP of 2.5 reflecting omnivory, and a TP of 3 reflecting higher carnivory. We calculated TP in two ways: (1) with glutamic acid representative of metazoan food webs and (2) with alanine, which has been recently identified for tracking protistan heterotrophy in pelagic food webs (Décima et al. 2017; Décima et al. 2020; Shea et al. 2023). We used phenylalanine as the source amino acid for both calculations. The TP of *Calanus* spp. was calculated using Equation 1.

$$\mathrm{TP}=\frac{\left(\delta^{15}N_{\text{Trophic}}-\delta^{15}N_{\mathrm{Phe}}\right)-\beta }{\mathrm{TDF}}+1$$

Where *Trophic* is either glutamic acid or alanine, *β* is the difference between the *Trophic* amino acid and *Phe* in primary producers (β_Glu_ = 3.4‰; β_Ala_ = 3.2‰), and *TDF* is the trophic discrimination factor, representing the enrichment in ^15^N_AA_ with each trophic step (TDF_Glu_ = 7.1‰; TDF_Ala_ = 4.5‰). β and TDFs were derived from laboratory experiments and field observations (Bradley et al. 2015; Décima et al. 2017; Décima et al. 2020; Shea et al. 2023). These pairings were determined a posteriori when samples had been analyzed and quantified. The protistan food web contribution was quantified following Décima et al. (2020), as the difference between the two TP estimates Equation 2.

(2) $$\mathrm{\Delta TP}={\mathrm{TP}}_{\mathrm{Ala}}-{\mathrm{TP}}_{\mathrm{Glu}}$$

We used an unpaired *t*‐test to check for differences in TP between the two TP calculations that is TP_Ala_ and TP_Glu_. To determine if there were spatial and interannual differences in *Calanus* spp. TP and DTP, we used a one‐way Analysis of Variance and Tukey's HSD post hoc tests between the three regions within the open water grouping and between each year (year was treated as a factor). The residuals of each ANOVA model were checked for normality to assess the model's fit, and where the data did not meet the assumptions of an ANOVA, Kruskal–Wallis tests were used.

## 3 Results

### 3.1 Seasonal and Interannual Environmental Variability

**FIGURE 3:**
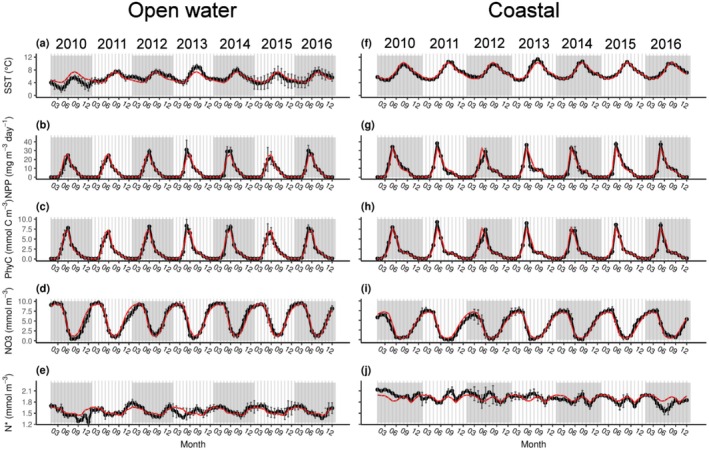
Time series of environmental conditions that influence the nitrogen isotope baseline in the open water (panels a, c, e, g and i) and coastal (panels b, d, f h and j) regions of the Barents Sea Opening between January 2010 and December 2016. The red line indicates the time‐series average for each month. N* was calculated from model derived NO_3_ and PO_4_ concentrations. Phytoplankton carbon (PhyC), net primary production (NPP) and SST were derived from satellite observations. Full details of the sources of these variables are in Data S2.

Sea surface salinity tracked known water masses, with fresher waters in the coastal region reflecting the Norwegian Coastal Current and more saline waters in the open water region reflecting the path of Atlantic water advected eastwards into the Barents Sea and northwards to produce the West Spitsbergen Current (Oziel et al. 2016; Figure S1). In the open water region, 2013 was the warmest year, with monthly averages being *~*2°C greater than the time‐series average between June and October (Figure 3a). NPP and total phytoplankton carbon were highest in 2013 in the open water region, with measurements up to 40 mg^−3^ day^−1^ and 10 mmol C m^−3^, respectively, during the bloom peak in May (Figure 3b,c). Nitrate concentrations reflected a lagged effect of NPP, with lowest concentrations occurring in July in 2013, 2 months after the NPP peak in May as the nitrate was depleted during bloom development (Figure 3d). In the open water region, N* was also higher than the time‐series average between June and October in 2013 (Figure 3e).

SST, NPP, and N* were elevated in the coastal region relative to the open water region (Figure 3), whilst nitrate concentrations were generally lower in the coastal region (Figure 3). NPP peaked in the open water region in June in 2010, 2011, and 2012, but then from 2013 onwards, NPP peaked in May at *~*30 mg^−3^ day^−1^ (Figure 3), whereas in the coastal region, NPP peaks of *~*35 mg^−3^ day^−1^ occurred in May, except for 2012, where a below‐average summer peak occurred in June (Figure 3).

**FIGURE 4:**
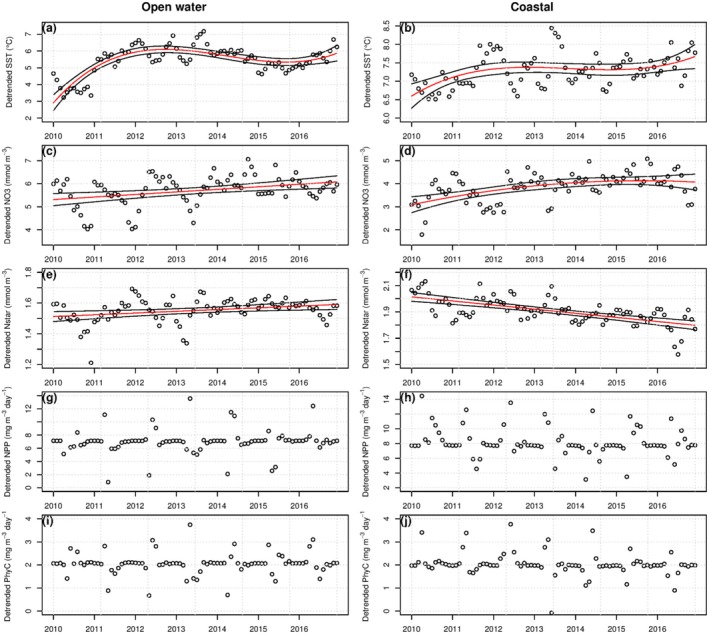
Temporal trends in (a & b) sea surface temperatures; (c & d) NO_3_; (e & f) N* (N star); (g & h) NPP; and (i & j) phytoplankton carbon. The trends have had the seasonal effect removed, therefore the *y*‐axes no longer represent absolute values, however the data shown are still in the units displayed in the *y*‐axis label. Linear models were fit for panels b, c, e and f. Non‐linear models were fit for panel a (a cubic polynomial) and d (quadratic polynomial) to capture the non‐linear nature of the temporal trends. Red lines show the model, the black lines show the upper and lower 95% confidence intervals. No trend was found for NPP (g & h) or phytoplankton carbon (i & j) so no line is shown. Model outputs are displayed in Table 2.

By employing the seasonal trend decomposition to extract the seasonal patterns of the environmental variables and then applying regression models, we were able to identify temporal trends. In the open ocean region, we observed a significant non‐linear trend in SST, which increased from 2010 to 2013. SST was similar in 2014 and 2015, but then increased again from 2016 onwards (Figure 4a; Table 2). A linear, but weaker, increasing trend was observed for SST in the coastal region (Figure 4b; Table 2). Weak but significant increases in open ocean nitrate (Figure 4c; Table 2) and N* (Figure 4e; Table 2) were observed, whereas a strong negative linear trend was observed for N* in the coastal region (Figure 4f; Table 2). Nitrate in the coastal ocean showed a non‐linear trend (Figure 4d; Table 2). No significant trends were observed for NPP or total phytoplankton carbon (Figure 4g–j; Table 2).

**TABLE 2:** Regression coefficients showing the temporal trends in environmental ocean characteristics from 2010 to 2016. Trends were determined using a seasonal trend decomposition to extract the seasonal component, and a linear or non‐linear model applied to the seasonality‐adjusted data. Polynomial 1, 2, and 3 refer to the 1st (linear regression), 2nd (quadratic polynomial) or 3rd (cubic polynomial) polynomial. Trends are visualised in Figure 4. Below the polynomial coefficients, the overall model coefficients are given (Adjusted *R* ^2^, *F*_degrees of freedom_ and *p* value). PhyC refers to total phytoplankton carbon.

	Open water					Coastal				
		Estimate	Std. Error	*t*	*p*		Estimate	Std. Error	*t*	*p*
SST	(Intercept)	5.44	0.06	89.01		(Intercept)	7.28	0.04	168.79	
	Polynomial 1	2.79	0.56	4.98	< 0.0001	Polynomial 1	1.47	0.40	3.71	0.0003
	Polynomial 2	−4.44	0.56	−7.93	< 0.0001					
	Polynomial 3	3.55	0.56	6.34	< 0.0001					
	Adj. *R* ^2^ = 0.6; *F*_3,80_ = 42.41; *p* = < 0.0001					Adj. *R* ^2^ = 0.18; *F*_3,82_ = 7.4; *p* = 0.0003				
NO3	(Intercept)	5.69	0.07	85.04		(Intercept)	3.84	0.06	65.11	
	Polynomial 1	2.05	0.61	3.34	0.001	Polynomial 1	2.64	0.54	4.88	< 0.0001
						Polynomial 2	−1.12	0.54	−2.07	0.04
	Adj. *R* ^2^ = 0.1; *F*_3,82_ = 11.17; *p* = 0.001					Adj. *R* ^2^ = 0.23; *F*_3,81_ = 14.04; *p* = < 0.0001				
N*	(Intercept)	1.55	0.01	188.84		(Intercept)	1.91	0.01	225.46	
	Polynomial 1	0.21	0.08	2.79	0.007	Polynomial 1	−0.58	0.08	−7.46	< 0.0001
	Adj. *R* ^2^ = 0.07; *F*_3,82_ = 7.79; *p* = 0.007					Adj. *R* ^2^ = 0.39; *F*_3,82_ = 55.63; *p* = < 0.0001				
NPP	(Intercept)	6.89	0.23	30.28		(Intercept)	7.88	0.31	25.22	
	Polynomial 1	3.03	2.09	1.45	0.151	Polynomial 1	−1.81	2.87	−0.63	0.53
	Adj. *R* ^2^ = 0.01; *F*_3,82_ = 2.10; *p* = 0.151					Adj. *R* ^2^ = −0.01; *F*_3,82_ = 0.52; *p* = 0.530				
PhyC	(Intercept)	2.05	0.05	40.08		(Intercept)	2.00	0.07	29.52	
	Polynomial 1	0.17	0.47	0.37	0.715	Polynomial 1	−0.69	0.62	−1.12	0.27
	Adj. *R* ^2^ = −0.01; *F*_3,82_ = 0.13; *p* = 0.715					Adj. *R* ^2^ = 0.003; *F*_3,82_ = 1.25; *p* = 0.266				

**FIGURE 5:**
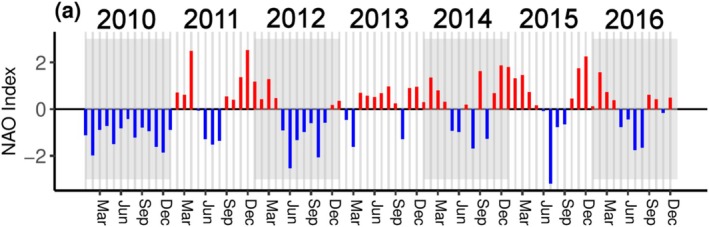
The monthly average North Atlantic Oscillation Index for 2010–2016. Red bars indicate a positive phase, blue bars indicate a negative phase.

Analysis of the North Atlantic Oscillation Index showed a negative phase during all of 2010, May to November of 2012, and sporadically later in the time series. After 2012, a positive NAO phase dominated much of the time series, with 32 of the 48 months having a positive average index (Figure 5).

### 3.2 Spatio‐Temporal Variability in Nitrogen Isotope Baseline

**FIGURE 6:**
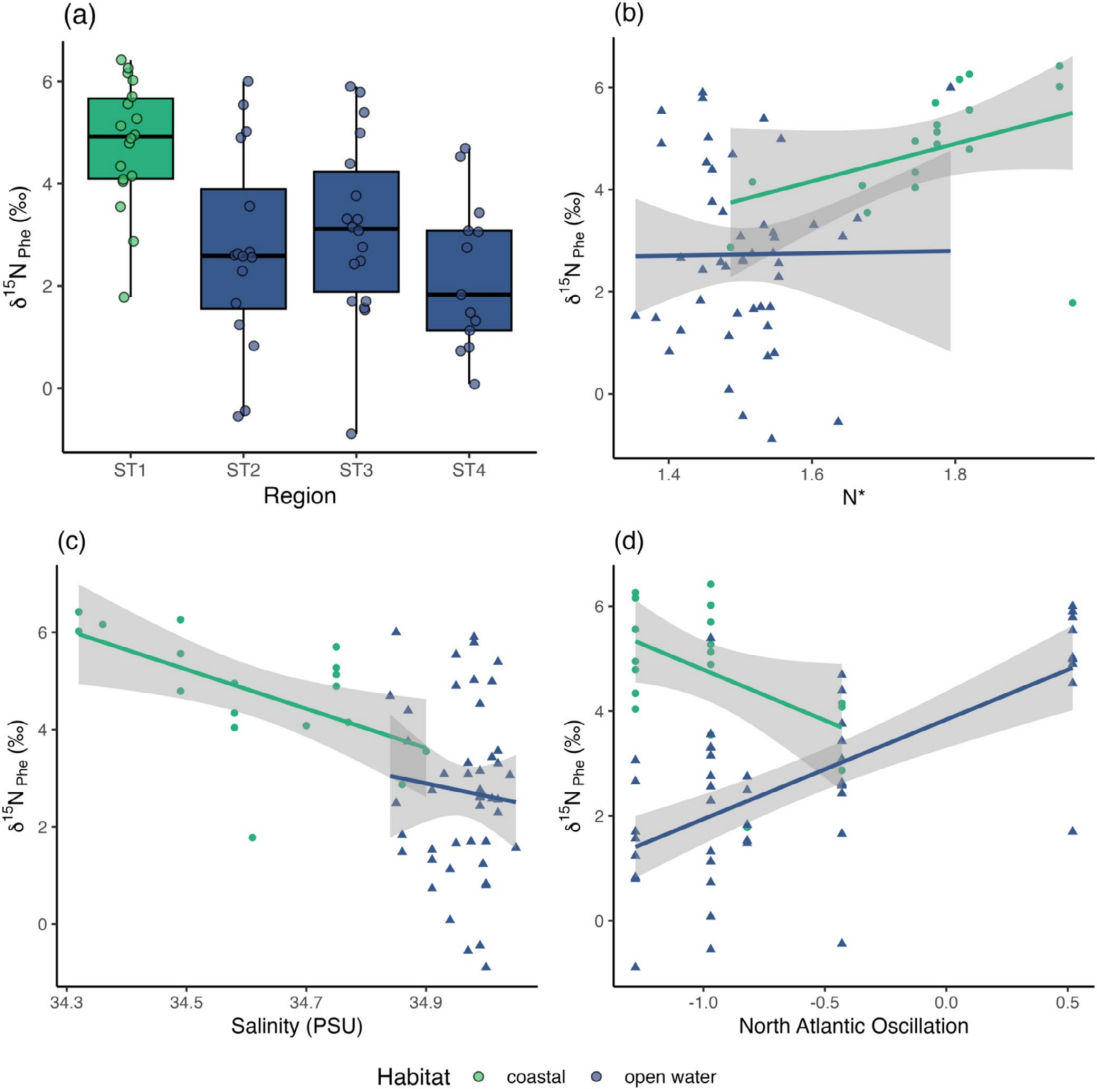
Spatial patterns in *Calanus* spp. δ^15^N_Phe_. (a) δ^15^N_Phe_ for the four regions. (b) correlation between δ^15^N_Phe_ and N*, (c) correlation between δ^15^N_Phe_ and salinity and (d) correlation between δ^15^N_Phe_ and the North Atlantic Oscillation, both the open water (blue) and coastal (green) regions are shown. Shaded areas in (b–d) show 95% confidence intervals around regression lines.

We found high spatial variability in the nitrogen isotope baseline, with δ^15^N_Phe_ ranging from −0.9‰ to 7.7‰ across the entire transect (Figure 6a). δ^15^N_Phe_ values at ST1 were on average 2.1‰ higher than in all other regions (Tukey's HSD; *p* values ranged from 0.003 to 0.01; *F* = 7.3; Figure 6a). We found strong evidence for a difference between the nitrogen isotope baseline in the coastal and open water regions (unpaired *t*‐test: *t* = 5.38, df = 43.68, *p* = < 0.0001; mean δ^15^N_Phe_ = 4.8‰ and 2.7‰ for coastal and open water regions, respectively, Figure 6a). Within ST1, samples closest to the Norwegian Coast had δ^15^N_Phe_ values of 6‰, whilst δ^15^N_Phe_ values of samples from 70.5°N to 72°N were between 3‰ and 5‰.

Across all years in the coastal region, salinity decreased and δ^15^N_Phe_ increased with proximity to the Norwegian Coast and the NCC. There was a strong relationship between coastal δ^15^N_Phe_ and both N* (Figure 6b) and salinity (Figure 6c) in the coastal region. δ^15^N_Phe_ values decreased by 5.2‰ for every 1 PSU increase in salinity (LR: Adj. *R* ^2^ = 0.27, *F*_1,59_ = 24.28, *p* < 0.0001), and by 7‰ for every 1 mmol m^−3^ increase in N* (LR: Adj. *R* ^2^ = 0.68, *F*_1,15_ = 34.4, *p* < 0.0001). There was no relationship between δ^15^N_Phe_ and N* (Figure 6b) or salinity (Figure 6c) in the open water regions, but there was a positive relationship between δ^15^N_Phe_ and the NAO index (LR: Adj. *R* ^2^ = 0.45, *F*_1,44_ = 37.9, *p* < 0.0001; Figure 6d), with δ^15^N_Phe_ values up to 6‰ in positive phases, and between 1‰ and 4‰ in negative phases (Figure 6d).

**FIGURE 7:**
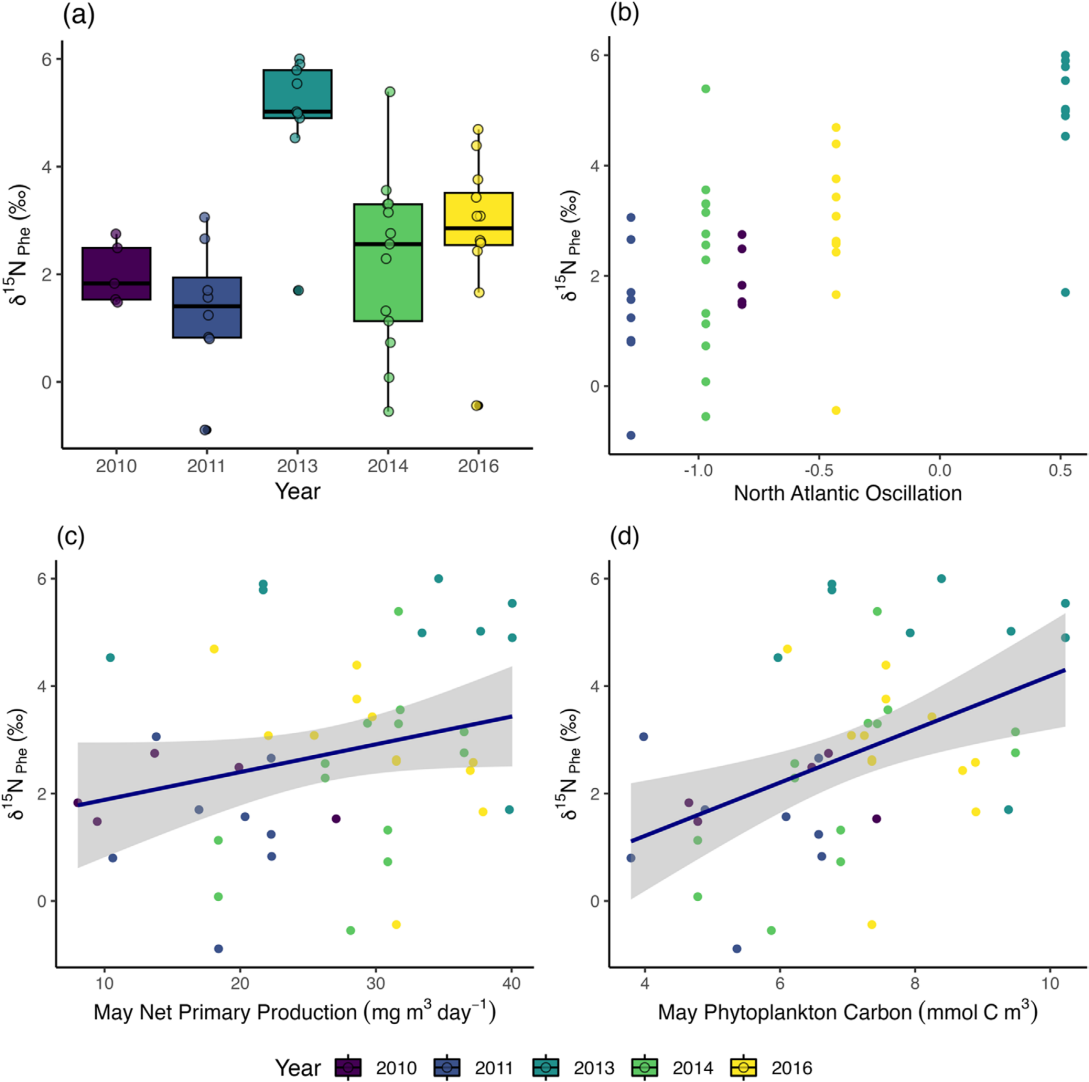
(a) Interannual patterns in open water *Calanus* spp. δ^15^N_Phe_. (b) Interannual correlation between open water *Calanus* spp. δ^15^N_Phe_ and North Atlantic Oscillation index in June, (c) Interannual correlation between open water *Calanus* spp. δ^15^N_Phe_ and NPP in May and (d) Interannual correlation between open water *Calanus* spp. δ^15^N_Phe_ and phytoplankton carbon in May. Shaded areas in (c & d) show 95% confidence intervals around regression lines.

In the coastal region, the limited sample size across years meant comparisons were only possible between 2011, 2014, and 2016, and δ^15^N_Phe_ values were marginally lower in 2016 (ANOVA: *F*_2,14_ = 3.77, *p* = 0.05, Table 3). However, in the open water regions, there was strong interannual variation in the nitrogen isotope baseline. δ^15^N_Phe_ values in 2013 averaged at 4.9‰ ± 1.3‰, which was 2.9‰, 3.5‰, 2.7‰ and 2.1‰ higher than the average for 2010 (Tukey's HSD; *p* = 0.001), 2011 (*p* < 0.0001), 2014 (*p* = 0.004) and 2016 (*p* = 0.009), respectively (Figure 7a). The year 2013 had the highest NAO values, and 2011 had the lowest values, and those 2 years had the biggest difference in δ^15^N_Phe_ values (Figure 7b) We found a positive relationship between open water δ^15^N_Phe_ and NPP in May (LR: Adj. *R* ^2^ = 0.17, *F*_1,62_ = 14.7, *p* = 0.0003; Figure 7c), and with total phytoplankton carbon in May (LR: Adj *R* ^2^ = 0.25, *F*_1,62_ = 21.8, *p* < 0.0001; Figure 7d).

**TABLE 3:** Interannual variation in δ^15^N_Phe_ (‰), TP_Glu_, TP_Ala_ and ΔTP for the coastal region (ST1) during 2011, 2014 and 2016. Mean values are given with standard deviation in parentheses. *n* is the number of samples.

Year	*n*	δ^15^N_Phe_ (‰)	TP_Glu_	TP_Ala_	ΔTP
2011	7	5.2 (0.8)	1.8 (0.2)	2.6 (0.2)	0.8 (0.3)
2014	7	5.3 (0.9)	2.0 (0.1)	3.0 (0.2)	1.1 (0.2)
2016	3	3.7 (0.7)	2.2 (0.2)	3.4 (0.3)	1.2 (0.1)

### 3.3 Spatio‐Temporal Variability in *C. finmarchicus* TP

There was variability in the extent of ^15^N enrichment between trophic amino acids measured in this study (ANOVA: *F*_4,234_ = 298.3, *p* < 0.0001, Tukey HSD *p* < 0.001 except Glu and Val; Figure S3). The amino acids Gly and Phe displayed lower ^15^N enrichment than the trophic amino acids; Gly was more enriched in ^15^N compared to Phe (ANOVA: *F*_1,98_ = 138.7, *p* < 0.0001, Figure S3). We have refrained from using Gly as a source amino acid here because of the current uncertainty in its classification; see McMahon and McCarthy (2016) and Nielsen et al. (2015).

**FIGURE 8:**
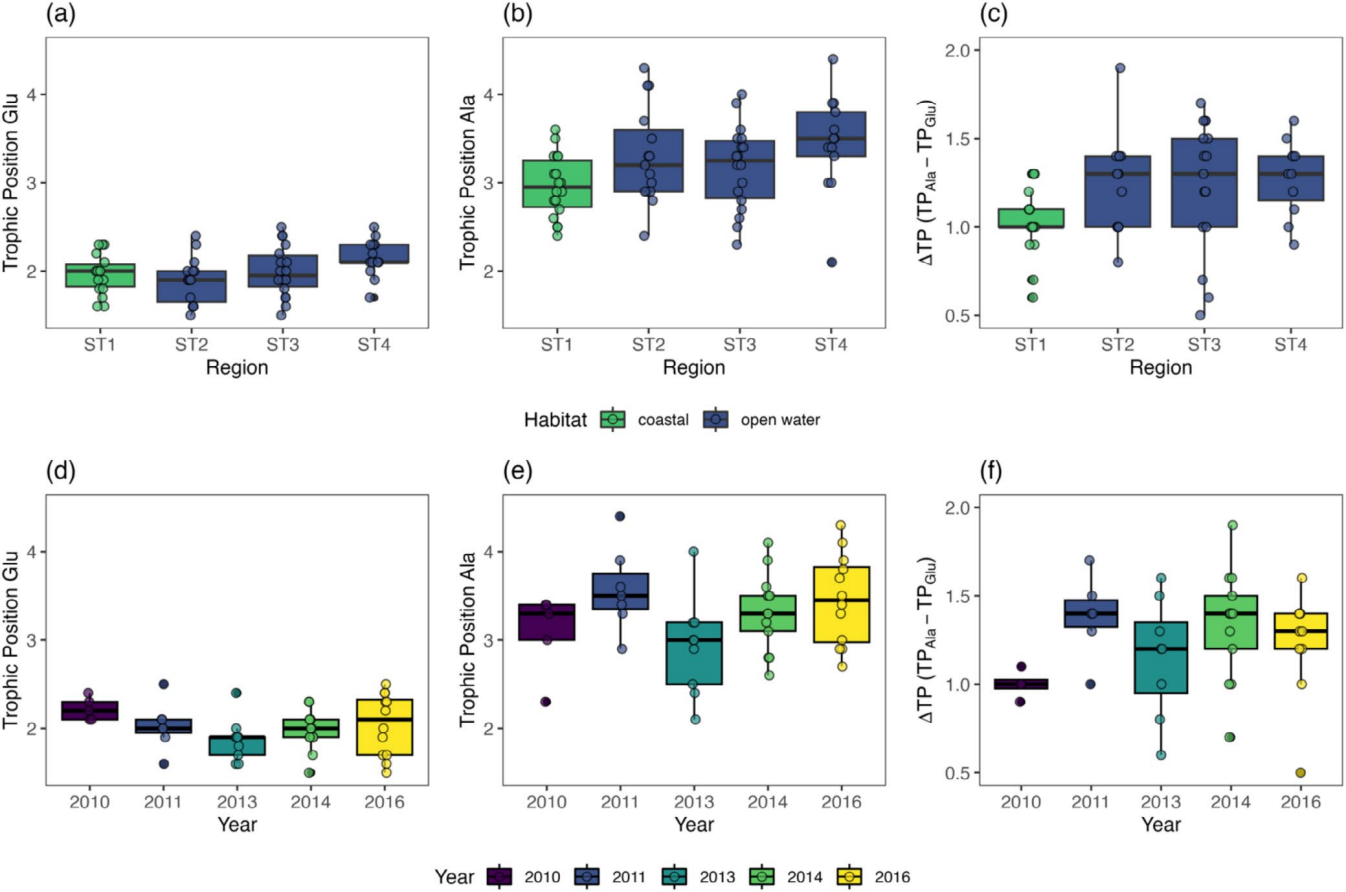
Spatial (a–c) and interannual (d–f; open water region only) variability in the trophic position estimates of *Calanus* spp. Green and blue boxes in a–c represent coastal and open water regions, respectively. Trophic position is calculated using the amino acids: Glutamic acid and phenylalanine (a, d); and alanine and phenylalanine (b, e). The difference between the two trophic position estimates gives the integrated contribution of protists to Calanus spp. trophic position (c, f).

TP_Ala_ was consistently higher than TP_Glu_ (*t*‐test: *t* = 17.35, df = 81.66, *p*‐value < 0.0001; Figure 8). *Calanus* spp. TP_Glu_ ranged from 1.5 to 2.5 in the metazoan food web, whereas *Calanus* spp. TP_Ala_, accounting for protistan heterotrophy, was higher, ranging from 2.1 to 4.4. The integrated protistan contribution (ΔTP) to *Calanus* spp. TP accounted for 0.2–2.5 TP (Figure 8).

In the metazoan food web (TP_Glu_) there was no difference between coastal or open water regions, but TP_Glu_ was marginally lower at ST2 compared to ST4 (ANOVA: *F*_1,62_ = 0.22, *p* = 0.64; and *F*_3,60_ = 2.65, *p* = 0.06, respectively; Figure 8). In contrast, TP_Ala_ (accounting for protistan heterotrophy) was lower in the coastal region at ST1 compared to the open water region (ST2—ST4) (ANOVA: *F*_1,62_ = 6.13, *p* = 0.02, between coastal and open water regions; and Tukey HSD *p* = 0.03 across the transect; Figure 8). The integrated protistan contribution (ΔTP) to TP of *Calanus* spp. was lower in the coastal region compared to the open water region (Kruskal–Wallis: *p* = 0.03, between regions and across the transect; Figure 8).

In the coastal region, the limited sample size across years meant comparisons were only possible between 2011, 2014, and 2016. TP_Glu_ was lower in 2011 compared to 2014 and 2016 (mean TP_Glu_ = 1.8, 2.0, 2.2, respectively, ANOVA, *F*_2,14_ = 7.46, *p* = 0.006; Table 3). Similarly, TP_Ala_ was lower in 2011 compared to 2014 and 2016 (mean TP_Ala_ = 2.6, 3.1, 3.4, respectively, ANOVA, *F*_2,14_ = 19.2, *p* < 0.0001; Table 3). ΔTP was marginally lower in 2011 compared to 2014 and 2016 (mean ΔTP = 0.8, 1.1, 1.2, respectively, ANOVA, *F*_2,14_ = 3.54, *p* = 0.06; Table 3). In the open water region, a full comparison across the time series (2010–2016) was possible (Figure 8d–f). TP_Glu_ did not differ between years (ANOVA, *F*_4,41_ = 1.51, *p* = 0.22; Figure 8d). TP_Ala_ was marginally lower in 2013 (mean TP_Ala_ = 2.9, ANOVA, *F*_4,41_ = 2.44, *p* = 0.06; Figure 8e). ΔTP was marginally lower in 2010 (mean ΔTP = 0.8, ANOVA, *F*_4,41_ = 3.01, *p* = 0.02; Figure 8f).

## 4 Discussion

The nitrogen isotope baseline (δ^15^N_Phe_) in the Barents Sea Opening varied in space and time. Spatial variation in δ^15^N_Phe_ could be attributed to the seasonal timing of the bloom and maxima in NPP between different water masses along the transect. On the other hand, temporal variation, which we expected to reflect greater inflows of Atlantic water in certain years, was related to NPP and driven by interannual fluctuations in the North Atlantic Oscillation index. Whilst the temporal variation in δ^15^N_Phe_ tracked NPP, the TP_Glu_ of *Calanus* spp. did not increase with decreasing NPP as we hypothesised. Instead, an average TP_Glu_ of 2.2 ± 0.2 was observed throughout the entire time series. We noted an important contribution of protistan microzooplankton (TP_Ala_ and ΔTP) to the diet of *Calanus* spp., which varied between coastal and open water regions and interannually, potentially driven by gradients in NPP.

The nitrogen isotope baseline revealed the influence of local water masses in each region along the transect. The influence of the fresher Norwegian Coastal Current in the coastal ST1 region was evident due to higher δ^15^N_Phe_ values in this region (by 2‰) relative to the open water region. We also observed a positive correlation between δ^15^N_Phe_ and N*, with higher N* reflecting waters with excess nitrate relative to phosphate, generated by processes such as nitrogen fixation, atmospheric deposition of nitrogen, or riverine influence (Gruber and Sarmiento 1997). However, these processes typically introduce more ^14^N, creating isotopically lighter nitrate available for assimilation by primary producers. Instead, the driver of the increase in δ^15^N_Phe_ might be due to nutrient dynamics and phytoplankton fractionation specifically in the coastal region. In May in the coastal region, NPP was higher (up to 40 mg m^3^ day^−1^), and surface nitrate was lower relative to the open water region. Indeed, δ^15^N of nitrate dissolved in seawater is elevated in coastal waters (6‰–8‰) relative to open water (< 5‰; Tuerena et al. 2021b), supporting our observations. Despite low nitrate concentrations, higher N* values in the coastal region suggest nitrate is in excess, thus not limiting phytoplankton growth. The preferential assimilation of ^14^N in nitrate by phytoplankton that occurs in non‐nitrate‐limited conditions leaves the surface waters more enriched in ^15^N, whereas in nitrate‐limited environments, there is assimilation of all available N from nitrate, which reduces the fractionation effect (Buchanan et al. 2021; Mahaffey et al. 2004; Tuerena et al. 2021a). We postulate that in June, phytoplankton take up the heavier nitrate left in the surface waters (Figure 1), which is then transferred to copepods during grazing, resulting in the increased δ^15^N_Phe_ observed in the coastal region.

Where spatial variability of δ^15^N_Phe_ was driven by the timing and intensity of the phytoplankton bloom, interannual variability was associated with changes to NPP and phytoplankton carbon, driven by variability in the NAO. There was a positive correlation between open water δ^15^N_Phe_ and the NAO. This contrasted with our hypothesis that δ^15^N_Phe_ would be lower with a positive NAO phase. The δ^15^N of phenylalanine is highly correlated with δ^15^N of nitrate dissolved in seawater (de la Vega et al. 2021a). Nitrate is supplied to the Arctic from the Pacific ocean through the Bering Sea and from the North Atlantic ocean through the Barents Sea and eastern Fram Strait (Torres‐Valdés et al. 2013). Higher rates of nitrogen fixation reduce surface δ^15^N‐_NO3_ in the Atlantic Ocean compared to the Pacific Ocean, where water column denitrification enriches δ^15^N‐_NO3_ Atlantic δ^15^N‐_NO3_ = 5.5‰; Pacific δ^15^N‐_NO3_ = 6.7‰; (Rafter et al. 2019). Since a positive phase of the NAO causes a stronger inflow of Atlantic water into the Barents Sea Opening (Delworth et al. 2016), with lower δ^15^N‐_NO3_, we expected δ^15^N_Phe_ to reflect this lower Atlantic‐influenced δ^15^N‐_NO3_ in more positive NAO phases. Instead, we observed a significant enrichment in δ^15^N_Phe_ in years with a more positive NAO phase, particularly in 2013. Positive NAO phases increase air temperature and SST in the Barents Sea, which stimulates NPP in the Arctic (Krumhardt et al. 2020), as was observed in 2013. An enrichment in δ^15^N_Phe_ values occurred where productivity was high, which has also been observed in the Fram Strait (Tuerena et al. 2021). We suggest this is due to a preferential uptake of ^14^N by phytoplankton in May, leaving more ^15^N in the surface waters that was taken up by phytoplankton in June, which is subsequently assimilated by zooplankton with a turnover time of 15 days (Mayor et al. 2022). This effect was strongest in 2013, where δ^15^N_Phe_ values were between 2‰ and 3‰ higher compared to other years, which is comparable to the disparity in δ^15^N_Phe_ between the coastal and open water regions.

*Calanus* spp. TP inferred from glutamic acid suggested a solely herbivorous diet consisting exclusively of phytoplankton (TP_Glu_ = 2). TP inferred from alanine indicated a contribution of protistan microzooplankton to the diet with (TP_Ala_ = 3–4), with the ΔTP indicative of a protistan microzooplankton contribution equivalent to 1–2 TP. Solely relying on glutamic acid would lead to an underestimation of *Calanus* spp. TP by omitting a key prey item, as shown previously by Gutiérrez‐Rodríguez et al. (2014), Décima et al. (2017), Décima et al. (2020), Shea et al. (2023). We hypothesised that lower NPP and total phytoplankton carbon would lead to higher *Calanus* spp. TP as they utilise a more omnivorous feeding strategy to compensate for a reduction in phytoplankton food availability. NPP was higher in the coastal region where TP_Ala_ and ΔTP of *Calanus* spp. was lower (in some cases by 1TP) suggesting a lower contribution of protistan microzooplankton to their diets and potentially a higher contribution of phytoplankton (Figure 8b,c). Whilst we did not observe any temporal trends in NPP and phytoplankton carbon for the coastal or open water regions, *Calanus* spp. did display interannual variation in trophodynamics. In the coastal region TP_Glu_, TP_Ala_ and ΔTP were lower in 2011 suggesting a decrease in protistan microzooplankton in the diet at this time. In the open water region TP_Glu_ was consistent across the period (*~*2), whilst TP_Ala_ and ΔTP were lower in 2013, again suggesting a decreased contribution of protistan microzooplankton to the diet. Indeed TP_Glu_ infers a signal of consistent herbivory as the TP of *Calanus* spp. across the entire study area and time series was 2.2 ± 0.2 and coupled with relatively high δ^15^N_Phe_ suggests feeding on nitrate fuelled phytoplankton (Décima et al. 2020). Similarly, consistent TP_Glu_ have been observed in copepods at Station ALOHA in the Pacific (Hannides et al. 2009). *C. finmarchicus* is known to be solely herbivorous in the high Arctic depending on prey composition (Søreide et al. 2008), and both *C. finmarchicus* and *C. glacialis* have retained mostly herbivorous feeding during periods of high algal biomass (Ohman and Runge 1994; Søreide et al. 2008).

TP_Ala_ used here demonstrates that omnivory, and in particular reliance on protistan microzooplankton, plays an important role in *Calanus* spp. diets. Interannual variation in TP_Ala_ suggests flexibility in the diet of *Calanus* spp. that may be linked to prey availability. Omnivory has been observed in *C. finmarchicus* populations in the Arctic (Castellani et al. 2008; Choi et al. 2020; Leiknes et al. 2014; Søreide et al. 2008). *C. finmarchicus* is highly plastic in terms of both morphology and ecology, depending on their development, which relates to their physiological ‘comfort zone’ (Trudnowska et al. 2020). These differences in feeding ecology may be down to body size, life stage, and their physical ability to consume larger food items. Vertical migration of *Calanus* and within downwelling mesoscale eddies common to the region (Atadzhanova et al. 2018) expose *Calanus* to a sub‐optimal food environment in comparison to feeding conditions at the surface. Therefore, omnivorous behaviour may also be in response to this reduction in phytoplankton availability at depth.

Dietary flexibility implies that healthy populations of *Calanus* spp. could be maintained in the sub‐Arctic Atlantic despite changes to food availability, although this could be compounded by their lack of ability to efficiently consume prey items below 5 μm in size (Hansen et al. 1994). High abundances of *Calanus* should benefit tertiary feeders that predate on them, providing their lipid stores are not compromised by a change in diet. However, increases in omnivory would increase food chain length and reduce the total biomass within the food web due to inefficient energy transfer between trophic levels (Baumann 1995). A long‐term change in food chain length has already been observed in the high Arctic Canadian Archipelago, which was driven by alterations to zooplankton feeding complexity (de la Vega et al. 2021b), demonstrating that these processes are already influencing pelagic food webs.

The dietary flexibility of *Calanus* spp. in the Barents Sea demonstrated using TP_Glu_ and TP_Ala_ has implications for the use of copepods as an isotopic baseline in food web studies in the region, where *Calanus* is commonly assumed to occupy TP 2 (Hansen et al. 2012; Hobson and Welch 1992; Nilsen et al. 2008). We have shown using TP_Ala_ and ΔTP that protistan microzooplankton make an important contribution to the diet of *Calanus* spp. in the Barents Sea. Solely relying on TP_Glu_ would lead to an underestimation of *Calanus* spp. TP as TP_Glu_ does not account for protistan microzooplankton in the diet. Relying on TP_Glu_ or assuming a TP of 2 in *Calanus* could also result in an underestimation of TP in higher predators (Hussey et al. 2014), which has consequences for the use of food chain metrics in ecosystem management (McDonald‐Madden et al. 2016).

## 5 Conclusions

Our findings highlight the power of amino acid isotopes in detecting the impact of environmental variability on food web dynamics, specifically for copepods, at regional scales in the Barents Sea Opening. Spatial differences in the δ^15^N_Phe_ were observed, with the coastal region being highly enriched relative to the open water region, providing evidence for different nutrient dynamics between these areas given their correlation with N*. Highly enriched δ^15^N_Phe_ in 2013 in the open water regions was driven by a more positive NAO phase that brought in warmer SST and increased NPP. We have shown that environmental variability has little influence on the TP of *Calanus* spp. as determined by TP_Glu_, whereas the environment exerted influence over the nitrogen isotope baseline, which varied significantly over both spatial and interannual time scales. TP_Ala_ and ΔTP demonstrated the role of protistan microzooplankton in the diets of *Calanus* spp., exhibiting spatial variability potentially linked to NPP and phytoplankton availability. TP_Glu_ did not vary interannually, suggesting that *Calanus* spp. is able to maintain a high degree of herbivory in their diets, whilst interannual variation in TP_Ala_ suggests flexibility in the consumption of protistan microzooplankton potentially offering an alternative food source when phytoplankton availability becomes limited. As the Barents Sea continues to undergo physical and biological change, we suggest that the ability of *Calanus* spp. to maintain a high degree of herbivory alongside consumption of protistan microzooplankton could (assuming that their diet quality remains the same) provide stability to the rest of the food web given their important role as transferrers of energy from lower to higher trophic levels.

## Author Contributions

**Elliott Price:** conceptualization (equal), data curation (lead), formal analysis (lead), investigation (equal), methodology (lead), project administration (equal), visualization (lead), writing – original draft (lead), writing – review and editing (lead). **Claire Mahaffey:** conceptualization (equal), funding acquisition (lead), investigation (equal), project administration (equal), resources (equal), supervision (equal), writing – original draft (equal), writing – review and editing (equal). **Rowena Stern:** conceptualization (equal), funding acquisition (equal), investigation (equal), project administration (equal), resources (equal), supervision (equal), writing – original draft (supporting), writing – review and editing (supporting). **Claudia Castellani:** conceptualization (supporting), investigation (equal), supervision (equal), writing – review and editing (supporting). **Rachel M. Jeffreys:** conceptualization (equal), funding acquisition (equal), investigation (equal), project administration (equal), resources (equal), supervision (lead), writing – original draft (equal), writing – review and editing (equal).

## Conflicts of Interest

The authors declare no conflicts of interest.

## Supporting information


Data S1

## Data Availability

Amino acid isotope data is stored and managed by the British Oceanographic Data Centre (BODC) the access number is BODC ULV230047. Further inquiries can be made to: enquiries@bodc.ac.uk. All hydrography data was downloaded from open‐source datasets, the details of which can be found in the Supporting Information.
