# Supplementary material for: Dietary Flexibility of Calanoid Copepods in the Sub‐Arctic Atlantic: The Role of Protistan Microzooplankton
Source: Ecol Evol. 2025 Mar 16;15(3):e71080. doi: 10.1002/ece3.71080 (PMC11911133; doi:10.1002/ece3.71080)
Supplement: Supplementary file 1 — Data S1 [file ECE3-15-e71080-s001.docx]

# **Supplementary Materials**


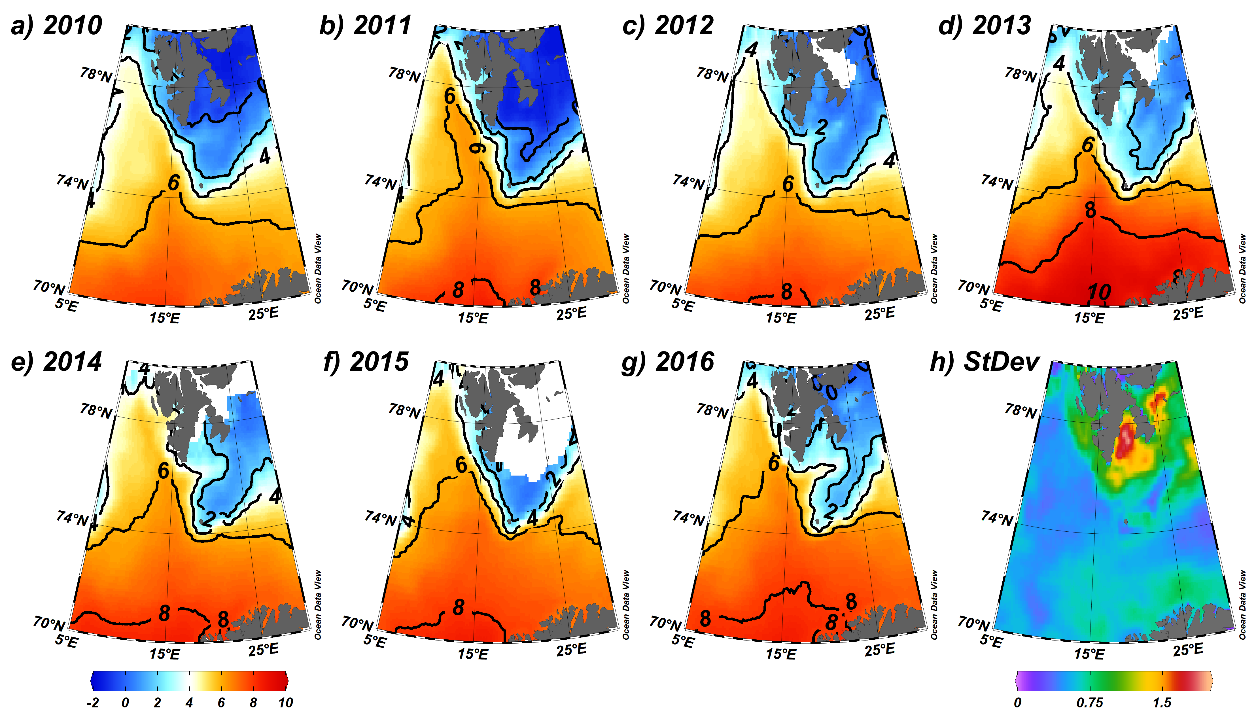


**Figure S1 Average June sea surface temperature (°C) (a-g) and standard deviation of these averages (h) in the Barents Sea Opening. Black lines represent the two-degree isoclines. White regions denote missing data.**

**
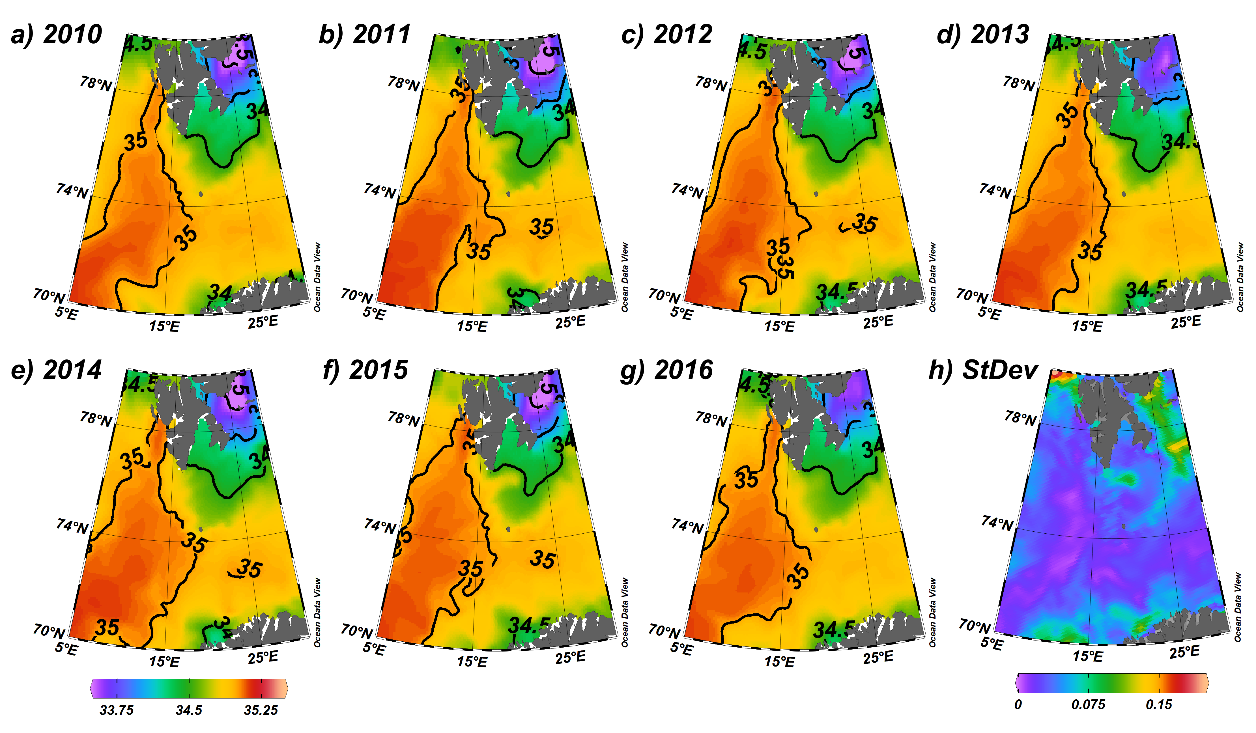
Figure S2 Average June sea surface salinity (PSU) (a-g) and standard deviation of these averages (h) in the Barents Sea Opening. Black lines represent the 0.5-PSU isoclines. White regions denote missing data**


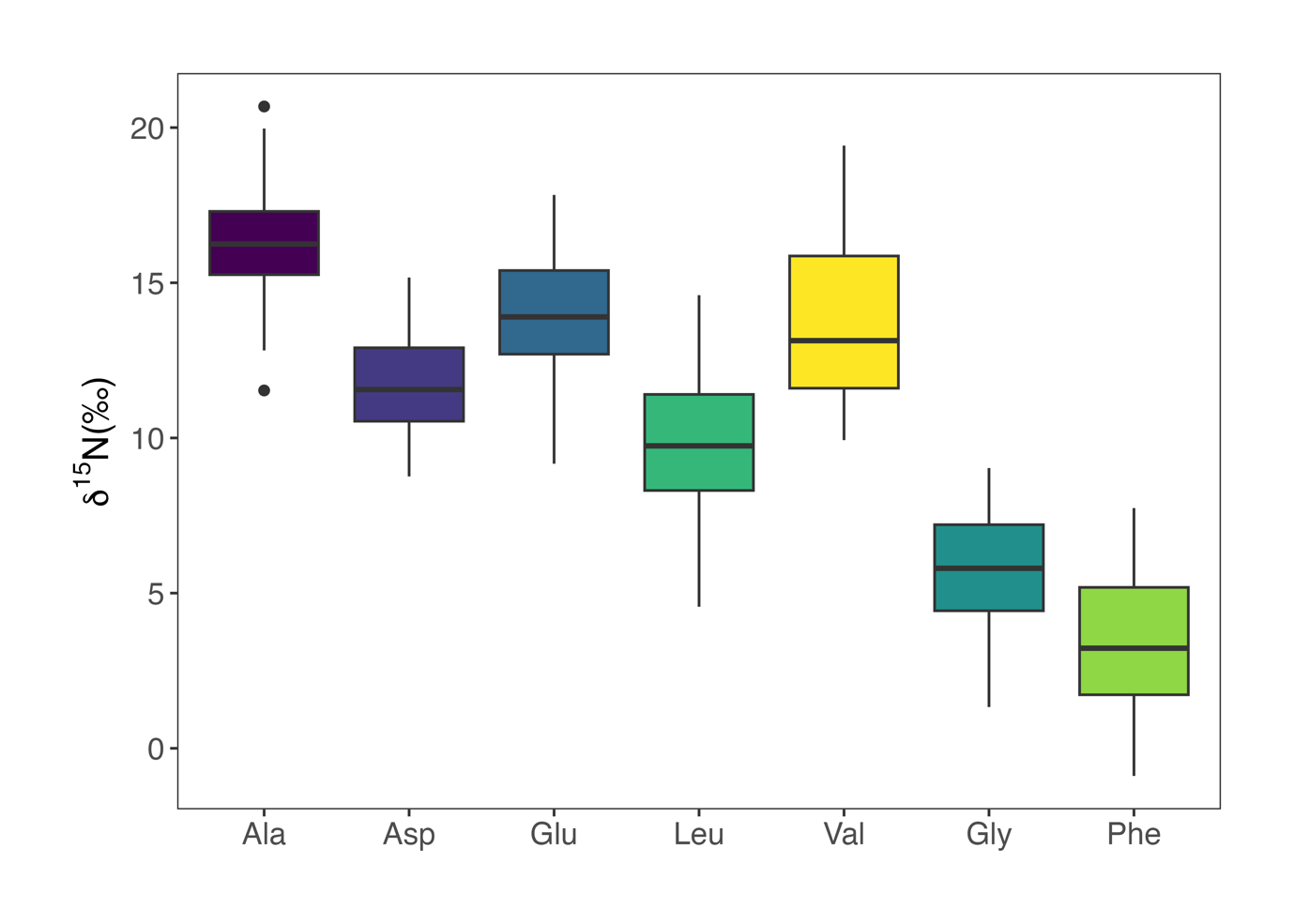


**Figure S3 Variability in δ^15^N for individual amino acids in *Calanus* spp. across the whole data set.**

**Table S1 Instrument Precision and Accuracy**

**Data S2**

**Environmental dataset collection and limitations**

Sea surface temperature data spanning from 2010 to 2016 was sourced from the OSTIA project. The OSTIA product is an interpolation of microwave and infrared satellite measurement coupled with in-situ observations at a grid resolution of 1.5 km x 5.6 km for the Barents Sea (Donlon et al., 2012). Surface salinity was sourced from the TOPAZ4 system produced by the Arctic Marine Forecasting Centre (MFC) of the MyOcean project (http: //www.myocean.eu.org). The TOPAZ4 system uses the Hybrid Coordinate Ocean Model (HYCOM v2.2.) and assimilates ocean and ice observations on a large scale using an ensemble Kalman filter (EnKF) to provide salinity estimates at a resolution of 12.5 km x 12.5 km (Sakov et al., 2012). Average monthly surface temperature and salinity datasets for June in our study region were downloaded from https://marine.copernicus.eu/.

Nutrients and phytoplankton carbon were extracted from hindcast simulations performed using the Pelagic Interactions Scheme for Carbon and Ecosystem Studies version 2 (PISCES-v2) biogeochemical model, attached to the Nucleus for European Modelling of the Ocean version 4.0 (NEMOv4) general ocean circulation model (Aumont et al., 2015). Horizontal model resolution varied between ~0.5° at the equator and poles and 2° in the subtropics, while vertical resolution varied between 10 and 500 metres thickness over 31 levels. All output on the NEMO curvilinear, tripolar grid was re-gridded onto a regular 1° by 1° horizontal grid prior to data extraction. The main deficiencies of the model (accuracies of oxygen minimum zones, silicic acid in the Southern Ocean, and seasonal chlorophyll cycles in the subarctic Pacific Ocean (Aumont et al., 2015), have little relevance to the content of this study. We used the June monthly average for years 2010 to 2016 and extracted values from the closest grid cells where samples were taken. Data was accessed from Copernicus (<https://catalogue.marine.copernicus.eu/documents/PUM/CMEMS-GLO-PUM-001-029.pdf>)

Monthly means of surface net primary production estimates were sourced from the satellite-observation Global Ocean (Copernicus-GlobColour) product, which uses an algorithm sourced from Antoine & Morel (1996) and Morel (1997). Resolution is at 4km x 4km. Further details can be found in the Product User Manual (<https://catalogue.marine.copernicus.eu/documents/PUM/CMEMS-OC-PUM-009-ALL.pdf>). Phytoplankton carbon estimates were taken from the CMEMS global biogeochemical multi-year hindcast

The North Atlantic Oscillation index was sourced from the Nation Oceanic and Atmospheric Administration, at <https://www.ncdc.noaa.gov/teleconnections/nao/>. It is calculated as the surface sea level pressure difference between the subtropical high and subpolar low, with a positive phase reflecting below normal pressure in high latitudes.

The denitrification index (N*) was calculated using equation from Deustch et al., (2001) as follows:

N* = (N – 16P) + 2.90 μmol kg^-1^.

Where N and P are surface concentrations of nitrate and phosphate, respectively. The 2.90 is the global offset so that average global N* is equal to 0, which is a modification from of the original equation by Gruber and Sarmiento (1997).

The environmental datasets used in this study to understand the interaction between the physical and chemical properties of the ocean and the isotopic ecology of *Calanus* were derived from remote-sensing and modelled sources as outlined above. Such data are inherently limited in comparison to in-situ measurements due to uncertainty in relationships between variables in which the characteristic of interest is derived, and more practical difficulties in measurements such as cloud cover which can increase the occurrence of missing values. The OSTIA product, used to measure global sea surface temperatures, has an accuracy of 0.57 °C when compared to in situ-measurements (Donlon et al., 2012). Salinity was sourced from the TOPAZ4 system, this system has been subject to numerous validation checks that have shown its validity, particularly for our study area given it correctly determines ocean circulation in the Nordic seas which is a vital factor for modelling sea ice and salinity in the arctic (Sakov et al., 2012). Nutrient concentrations were sourced from the Pelagic Interactions Scheme for Carbon and Ecosystem Studies version 2 (PISCES-v2) biogeochemical model, attached to the Nucleus for European Modelling of the Ocean version 4.0 (NEMOv4), which has been found to produce consistent measures of surface nutrients, chlorophyll and mesozooplankton distributions. Net primary production, total phytoplankton carbon and chlorophyll was sourced from the Copernicus-GlobColour product. When compared to in-situ measurements across the global ocean there was strong agreement with remote sensed measurements for phytoplankton carbon (diatoms, dinoflagellates, haptophytes and green algae: R^2^ = 0.61 to 0.62; slopes = 0.7 to 0.95) and chlorophyl-a (R^2^ = 0.71 to 0.75, slopes = 0.97 to 0.99), but a lack of in-situ for primary production means that NPP measures are less-well validated and could have brought some uncertainty into the NPP values used (Garnesson et al., 2022).

**References**

Antoine, D. and Morel, A.: Oceanic primary production: 1. Adaptation of a spectral light-photosynthesis model in view of application to satellite chlorophyll observations, Global Biogeochem. Cycles, 10(1), 43–55, doi:10.1029/95GB02831, 1996.

Aumont, O., Ethé, C., Tagliabue, A., Bopp, L. and Gehlen, M.: PISCES-v2: An ocean biogeochemical model for carbon and ecosystem studies, Geosci. Model Dev., 8(8), 2465–2513, doi:10.5194/gmd-8-2465-2015, 2015.

Deutsch, C., Gruber, N., Key, R. M., Sarmiento, J. L. and Ganachaud, A.: Denitrification and N2 fixation in the Pacific Ocean, Global Biogeochem. Cycles, 15(2), 483–506, 2001.

Donlon, C. J., Martin, M., Stark, J., Roberts-Jones, J., Fiedler, E. and Wimmer, W.: The Operational Sea Surface Temperature and Sea Ice Analysis (OSTIA) system, Remote Sens. Environ., 116, 140–158, doi:10.1016/j.rse.2010.10.017, 2012.

Garnesson, P., Mangin, A., & Bretagnon, M. (2022). *Ocean Colour Production Centre, Satellite Observation, Copernicus-GlobColour Products, Quality Information Document*. Copernicus Marine Service.

Gruber, N. and Sarmiento, J. L.: Global patterns of marine nitrogen fixation and denitrification, Global Biogeochem. Cycles, 11(2), 235–266, 1997.

Morel, A.: Optical properties of oceanic case 1 waters revisited, in Ocean Optics XIII (Vol. 2963, pp. 108–115), International Society for Optics and Photonics, February 1997.

Sakov, P., Counillon, F., Bertino, L., Lister, K. A., Oke, P. R. and Korablev, A.: TOPAZ4: An ocean-sea ice data assimilation system for the North Atlantic and Arctic, Ocean Sci., 8(4), 633–656, doi:10.5194/os-8-633-2012, 2012.
